# The COVID‐19 pandemic has not changed stage at presentation nor treatment patterns of head and neck cancer: A retrospective cohort study

**DOI:** 10.1111/coa.14048

**Published:** 2023-03-16

**Authors:** Kelten Clements, Anna Cowell, Gillian White, William Flynn, David I. Conway, Catriona M. Douglas, Claire Paterson

**Affiliations:** ^1^ School of Medicine, Dentistry and Nursing University of Glasgow Glasgow UK; ^2^ Beatson West of Scotland Cancer Centre Glasgow UK; ^3^ Department of Otolaryngology – Head and Neck Surgery Glasgow Royal Infirmary Glasgow UK; ^4^ Glasgow Head and Neck Cancer (GLAHNC) Research Group Glasgow UK

**Keywords:** COVID‐19, COVID‐19 pandemic, head and neck cancer

## Abstract

**Objectives:**

To evaluate the impact of the COVID‐19 lockdown measures on HNC, by comparing the stage at presentation and treatment of HNC before and after the most severe COVID‐19 restrictions.

**Design:**

A retrospective cohort study.

**Setting:**

A regional cancer network serving a patient population of 2.4 million.

**Participants:**

Newly diagnosed patients with HNC between June and October 2019 (pre‐pandemic) and June and October 2021 (post‐pandemic).

**Main outcome measures:**

Symptom duration before diagnosis, stage at diagnosis, patient performance status (PS) and intent of treatment delivered (palliative vs. curative).

**Results:**

Five hundred forty‐five patients were evaluated—250 in the 2019 and 295 in the 2021 cohort. There were no significant differences in symptom duration between the cohorts (*p* = .359) or patient PS (*p* = .821). There were no increased odds of presenting with a late (Stage III or IV) AJCC cancer stage in 2021 compared with 2019 (odds ratio [OR] = 0.90; 95% confidence interval [CI]: 0.76–1.08); nor increased odds of receiving palliative rather than curative treatment in 2021 compared with 2019 (OR = 0.68; 95% CI: 0.45–1.03).

**Conclusion:**

The predicted stage shift to more advanced disease at the time of diagnosis of HNC due to the COVID‐19 pandemic has not been realised in the longer term. In keeping with this, there was no difference in symptom duration, patient PS, or treatment patterns between the 2019 and 2021 cohorts.

**Key points**

- The public health emergency caused by the coronavirus pandemic created major disruption to the National Health Service (NHS).
- The public health message was to not use the NHS, it is unknown how this has impacted on head and neck cancer (HNC).
- There is a lack of evidence as to the impact of the pandemic on HNC stage presentation, symptom duration and treatment intent.
- This retrospective study did not demonstrate a difference in symptom duration, stage, performance status or treatment intent pre‐ and post‐pandemic in a large cohort of HNC patients.
- The impact of the pandemic on survival has not been assessed yet.

## 1 INTRODUCTION

Healthcare services around the world have faced a unique set of challenges during the COVID‐19 pandemic. In the United Kingdom, the National Health Service (NHS) was put on an emergency footing in March 2020 to deal with an expected surge in COVID‐19 patients. In keeping with measures in several other countries, all non‐urgent surgery and outpatient clinic activity were suspended along with public health messaging for the population not to attend the health services unless absolutely necessary.^1^ This was accompanied with a rapid reduction in ‘urgent suspicion of cancer’ referrals from primary to secondary care.^2^ The suspended elective services are essential to the early detection of cancer. Globally, COVID‐19 public health measures were used to limit the transmission of coronavirus, the most severe of which resulted in national ‘lockdowns’. These measures are also associated with a reduction in healthcare service use. Both these factors risked the timely diagnoses of head and neck cancer (HNC)^3^ being made, with modelling predicting a stage shift to more advanced disease when eventually diagnosed.^4^

While studies published to date have compared the pre‐lockdown period to cohorts during the first 2020 national lockdown,^5^ one might expect the full impact of the restrictions described above to be observed at a later time point. This is yet to be evaluated.

The aim of this study is to investigate the impact of the COVID‐19 public health measures on stage at diagnosis and treatment patterns of HNC after the most severe restrictions were lifted.

## 2 PATIENTS AND METHODS

This retrospective cohort study was conducted according to Strengthening the Reporting of Observational Studies in Epidemiology (STROBE) guidelines for cohort studies. It was approved by the MVLS college ethics committee of the University of Glasgow (Project no: 200210121) for the extraction and analysis of patients' clinical information.

### 2.1 Eligibility criteria

The time periods sampled were ‘pre‐pandemic’ (1 June to 31 October 2019) and ‘post‐pandemic’ (1 June to 31 October 2021). These dates were chosen to be after the rollout of the vaccination programme permitting the gradual lifting of national COVID‐19 public health measures (except social distancing and mask wearing).

### 2.2 Inclusion criteria

1. Patients with newly diagnosed HNC
2. HNC cases were defined using the International Classification of Disease for Oncology, 3rd edition (ICD‐O‐3)—nasopharynx (C11), oropharynx (C01, C09, C10), hypopharynx (C12, C13), lip and oral cavity (C00, C02–C06), salivary glands (C07, C08), larynx (C32, C10), sinonasal (C30, C31) and CUP (cancer of unknown primary) (C80).^6^

### 2.3 Exclusion criteria

1. Recurrent cancers
2. Head and neck haematological and cutaneous cancers
3. Thyroid cancer

### 2.4 Data collection

Patients were identified from the regional multidisciplinary team (MDT) database, which includes all patients newly diagnosed with HNC in the West of Scotland serving a patient population of 2.4 million. Data were collected from electronic clinical records.

Demographic data included: age at diagnosis, sex, history of smoking (current/former‐smoker or never smoker) or excess alcohol consumption (current/former alcohol excess or never alcohol excess) defined by UK government guidance as more than 14 units per week; symptom duration which was estimated by the clinician at time of referral as the number of weeks of red flag symptoms and categorised arbitrarily into <6 weeks, 6–12 weeks and >12 weeks; whether the type of referral was emergency or non‐emergency. Performance status (PS) was recorded via the Eastern Cooperative Oncology Group (ECOG) PS scale.

Patients' home postcodes were linked to the Scottish Index of Multiple Deprivation (SIMD) 2020 scores. SIMD 2020—an area‐based measure of socioeconomic deprivation—categorises geographical areas of Scotland using information from seven domains: income; employment; education; health; access to services; crime and housing. The SIMD 2020 score has five levels with each quintile containing 20% of Scotland's population, with SIMD 1 representing the most deprived areas and SIMD 5 the least deprived areas.^7^

Tumour characteristics data were extracted: stage at presentation (TNM classification of malignant tumours 8th edition and AJCC overall clinical stage); HPV status of oropharyngeal cancers and cancers of unknown primary (CUP); cancer subsite.

Treatment details: intent of treatment delivered (curative or palliative); modality delivered—BSC (best supportive care), palliative SACT (systemic anti‐cancer treatment), primary (C) RT ((chemo)radiotherapy), surgery alone, surgery + adjuvant (C)RT, IC (induction chemotherapy) + (C)RT.

### 2.5 Statistical analysis

Descriptive statistics of patient characteristics for both pre‐pandemic and post‐pandemic cohorts were calculated. Pearson's Chi‐squared tests were used to demonstrate association between the categorical variables of demographics, tumour and treatment characteristics and the Student *t* test was used for continuous variables. *p* Values of less than .05 were considered significant.

Explanatory factors that may affect presenting stage between the cohorts were evaluated using univariate analysis. Univariate analysis was performed with age, sex, SIMD, PS, history of smoking and alcohol excess.^8^ The Mann–Whitney *U* test was used for nominal dichotomous variables, Kruskal–Wallis *H* test for ordinal non‐dichotomous variables and Spearman correlation for continuous variables. Univariate and multivariate analysis using a binary logistic regression model was used to calculate the odds ratio (OR) and 95% confidence interval (CI) of presenting with late stage in 2021 compared with 2019, only the statistically significant covariates identified from the univariate analysis of explanatory variables were included in the multivariate analysis.

Changes in treatment intent patterns were evaluated in a similar way to stage using a univariate analysis and a multivariate analysis with a binary logistic regression model. All analyses were performed using SPSSv24 (IBM).

## 3 RESULTS

Five hundred forty‐five patients were included in this study, 250 patients in the 2019 cohort and 295 in the 2021 cohort. This was an 18% increase (*p* = .054) in the number of patients with a new diagnosis of HNC in 2021 compared with the equivalent time period in 2019.

### 3.1 Patient characteristics

Patient characteristics are shown in Table 1, they did not differ significantly between the two cohorts. Most of the patients with HNC lived in deprived communities, with 43% of patients being from the 20% most deprived areas. 78% had a history of smoking and 96% had a non‐emergency presentation. There were no significant differences in symptom duration between the cohorts (*p* = .359). However, there were significantly more patients with a history of excessive alcohol consumption in the 2021 cohort compared with 2019 (*p* = .023).

**TABLE 1:** Baseline patient characteristics.

	Number (%)			
	Year			
Characteristic	Total	2019	2021	*p* Value^a^ (Chi‐square value)
Patients	545	250	295	.054^b^ (3.72)
**Age**				.795^c^
Mean (±SD)	65 (11.6)	65 (12.1)	65 (11.2)	
Range	26–96	26–96	37–91	
**Sex**				.157^b^ (2.01)
Male	387 (71)	185 (74)	202 (68)	
Female	158 (29)	65 (26)	93 (32)	
**History of smoking**				.419^b^ (0.65)
Current/former smoker	427 (78)	192 (77)	235 (80)	
Never smoker	118 (22)	58 (23)	60 (20)	
**History of excessive alcohol**				**.023** ^b^ **(5.18)**
Current/former alcohol excess	209 (38)	83 (33)	126 (43)	
Never alcohol excess	336 (62)	167 (67)	169 (57)	
**SIMD**				.944^b^ (0.76)
5 (20% least deprived)	62 (11.4)	29 (11)	33 (11)	
4	51 (9.4)	23 (9)	28 (10)	
3	72 (13.2)	33 (13)	39 (13)	
2	124 (23)	53 (21)	71 (24)	
1 (20% most deprived)	236 (43.3)	112 (45)	124 (42)	
**ECOG PS**				.821^b^ (1.53)
0—Fully active	243 (45)	108 (43)	135 (46)	
1—Strenuous activity restricted	163 (30)	77 (31)	86 (29)	
2—Ambulatory >50% waking time	87 (16)	40 (16)	47 (16)	
3—Ambulatory <50% waking time	51 (9)	24 (10)	27 (9)	
4—Fully bed/chairbound	1 (0)	1 (0)	0 (0)	
**Symptom duration**				.359^b^ (2.05)
Not recorded	112 (21)	32 (13)	80 (27)	
<6 weeks	184 (34)	100 (40)	84 (29)	
6–12 weeks	108 (20)	51 (20)	57 (19)	
>12 weeks	141 (26)	67 (27)	74 (25)	
**Emergency presentation**				.777^b^ (0.080)
Non‐emergency	524 (96)	241 (96)	283 (96)	
Emergency	21 (4)	9 (4)	12 (4)	

Abbreviations: ECOG, Eastern Cooperative Oncology Group; PS, performance status; SIMD, Scottish Index of Multiple Deprivation.

a Hypothesis testing of significant differences between cohorts.

^b^ Chi‐squared test.

^c^ Student *t* test.

### 3.2 Tumour stage at diagnosis

Tumour characteristics of new HNC cases are shown in Table 2. There were no significant differences in TNM stage between the two cohorts with advanced T3/4 stage representing 49% of both the 2019 and 2021 (*p* = .981). Around 50% of patients had nodal involvement and 3% had metastases at the time of diagnosis, with no significant differences in N or M stage between the two cohorts. The only significant difference in stage was a higher proportion of tumours in the 2021 cohort were AJCC 8th edition Stage II (22%) compared with 2019 (14%). Overall AJCC staging did not differ significantly between the two cohorts (*p* = .135) with the most common presentation for both cohorts' Stage IV (35%), followed by Stage I (26%).

**TABLE 2:** Tumour characteristics of new HNC cases.

	Number (%)			
	Year			
Characteristic	Total	2019	2021	*p* Value^a^ (Chi‐squared value)
**Total**	541	250	291	
**Location**				.490 (6.44)
Sinonasal	3 (1)	2 (1)	1 (0)	
Nasopharynx	16 (3)	10 (4)	6 (2)	
Oral cavity	130 (24)	55 (22)	75(26)	
Oropharynx	147 (27)	77 (31)	70 (24)	
Hypopharynx	47 (9)	22 (9)	25 (9)	
Larynx	169 (31)	72 (29)	97 (33)	
Salivary gland	19 (4)	8 (3)	11 (4)	
CUP	10 (2)	4 (2)	6 (2)	
**TNM Stage**				
**T**				.981 (1.11)
T0	10 (2)	4 (2)	6 (2)	
T1	130 (24)	60 (24)	70 (24)	
T2	139 (26)	65 (26)	74 (25)	
T3	107 (20)	47 (19)	60 (21)	
T4	69 (13)	34 (14)	35 (12)	
T4a	66 (12)	32 (13)	34 (12)	
T4b	20 (4)	8 (3)	12 (4)	
***N***				.647 (1.66)
N0	270 (50)	127 (51)	143 (49)	
N1	119 (22)	59 (24)	60 (21)	
N2	124 (23)	52 (21)	72 (25)	
N3	28 (5)	12 (5)	16 (6)	
**M**				.298 (1.08)
M0	522 (97)	239 (96)	283 (97)	
M1	19 (3)	11 (4)	8 (3)	
**AJCC Stage**				.135 (5.56)
I	143 (26)	71 (28)	72 (25)	
II	100 (19)	36 (14)	64 (22)	
III	109 (20)	50 (20)	59 (20)	
IV^b^	189 (35)	93 (37)	96 (33)	
**Overall AJCC Stage**				.359 (0.84)
Early (AJCC Stage I or II)	243 (45)	107 (43)	136 (47)	
Late (AJCC Stage III or IV)	298 (55)	143 (57)	155 (53)	

*Note*: Four cases were excluded from the analysis of stage in the 2021 cohort as the primary tumour could not be assessed.

Abbreviation: AJCC, American Joint Committee on Cancer; CUP, cancer of unknown primary; HNC, head and neck cancer; TNM, tumour node metastases.

^a^ Hypothesis testing of significant differences between cohorts.

^b^ Includes AJCC stages IV, IVa, IVb and IVc.

Age, PS, SIMD, history of smoking and alcohol excess were identified as being predictive of increased AJCC stage on univariate analysis (Table S1), so were adjusted for in the binary logistic regression model. Each model predicted an increase in AJCC above the intercept model with statistical significance being *p* < .01. Model 1 did not adjust for any covariates, so only tested year against AJCC stage, model 2 adjusted for age and PS and model 3 adjusted for age, PS, SIMD, history of smoking and alcohol excess. All these models found that there were no increased odds of presenting with a late AJCC cancer stage in 2021 compared with 2019 (Table 3).

**TABLE 3:** Univariate and multivariate binary regression analysis of presenting with late AJCC stage in 2021 versus 2019.

	Model 1^a^		Model 2^b^		Model 3^c^	
Characteristic	OR (95% CI)	*p* Value	OR (95% CI)	*p* Value	OR (95% CI)	*p* Value
OR of late AJCC stage presentation	0.923 (0.779–1.095)	.359	0.926 (0.779–1.102)	.387	0.902 (0.756–1.077)	.253

Abbreviations: AJCC, American Joint Committee on Cancer; CI, confidence interval; ECOG, Eastern Cooperative Oncology Group; OR, odds ratio; PS, performance status; SIMD, Scottish Index of Multiple Deprivation.

^a^ Unadjusted.

^b^ Adjusted for age and ECOG PS.

^c^ Adjusted for age, ECOG PS, SIMD, smoking and alcohol.

### 3.3 Treatment patterns and treatment intent

A summary of the treatments received by patients in both cohorts is listed in Table 4. Overall there were no significant differences in the proportions of treatments provided in 2019 compared with 2021 (*p* = .302). There were also no significant differences in treatment intent (palliative 38% 2019 vs. 31% 2021; curative 62% 2019 vs. 69% 2021) between the two cohorts (*p* = .081).

**TABLE 4:** Descriptive statistics of treatment.

		Number (%)			
		Year			
Treatment		Total	2019	2021	*p* Value^a^ (Chi‐squared value)
Intent	Type				.302 (9.50)
Curative	Surgery alone	136 (25)	54 (22)	82 (28)	
	Surgery + RT	45 (8)	20 (8)	25 (9)	
	Surgery + CRT	11 (2)	7 (3)	4 (1)	
	IC + RT	2 (0)	0 (0)	2 (1)	
	IC + CRT	11 (2)	7(3)	4 (1)	
	RT alone	82 (15)	35 (14)	47 (16)	
	Primary CRT	74 (14)	33 (13)	41 (14)	
Palliative	Palliative SACT	37 (7)	17 (7)	20 (7)	
	BSC	147 (27)	77 (31)	70 (24)	
Overall treatment intent					.081 (3.04)
Palliative		184 (34)	94 (38)	90 (31)	
Curative		361 (66)	156 (62)	205 (69)	

Abbreviations: BSC, best supportive care; CRT, chemoradiotherapy; RT, radiotherapy; SACT, systemic anti‐cancer treatment.

^a^ Hypothesis testing of significant differences between cohorts.

Age, PS, history of smoking and alcohol excess were identified as being predictive of treatment intent on univariate analysis (*p* < .05) (Table S2). Each model predicted the odds of having palliative treatment above the intercept model with statistical significance being *p* < .01. Model 1 did not adjust for any covariates, so only tested year against treatment intent, model 2 adjusted for age and ECOG PS and model 3 adjusted for age, ECOG PS, history of smoking and alcohol excess. None of these models demonstrated increased odds of having palliative treatment in 2021 compared with 2019. Although not statistically significant, the odds of having palliative treatment were slightly reduced for 2021 compared with 2019, see Table 5.

**TABLE 5:** Univariate and multivariate binary logistic regression analysis of receiving palliative treatment in 2021 versus 2019.

	Model 1^a^		Model 2^b^		Model 3^c^	
Characteristic	OR (95% CI)	*p* Value	OR (95% CI)	*p* Value	OR (95% CI)	*p* Value
OR of palliative treatment	0.729 (0.51–1.04)	.08	0.683 (0.45–1.03)	.07	0.634 (0.42–0.96)	.03

Abbreviations: CI, confidence interval; OR, odds ratio; PS, performance status.

^a^ Unadjusted.

^b^ Adjusted for age and PS.

^c^ Adjusted for age, PS, smoking and alcohol.

## 4 DISCUSSION

During the COVID‐19 pandemic, healthcare services around the world reallocated resources to provide sufficient capacity to treat patients with the novel coronavirus.^9^ This disruption, coupled with COVID‐19 public health measures and associated reduction in healthcare service use, has led to concerns that HNC cases may have gone undetected, ultimately presenting later and with more advanced disease. This hypothesis has been reinforced by modelling studies predicting upstaging of cancer cases, with a resultant increase in morbidity and mortality.^4^

We identified 23 published observational studies comparing the stage of HNC cases of a 2019 cohort to a 2020 cohort. Of these studies, 16 (70%) reported that patients did not present with more advanced disease during the pandemic in 2020.^5^, ^10^, ^11^, ^12^, ^13^, ^14^, ^15^, ^16^, ^17^, ^18^, ^19^, ^20^, ^21^ Furthermore, analysis of national cancer registry data showed that the incidence of HNC during 2020 was similar to what was observed in 2019, was in line with long term trends, and that patients were not presenting with more advanced disease during the pandemic.^22^ Our study reinforces these findings but also indicates that the pandemic is unlikely to have caused longer‐term changes to disease stage or treatment. The effects we captured during our sample were not the immediate disruptive effect of lockdowns, but rather the potential longer‐term implications these restrictions may have had due to delays in presentation. For the first time, we demonstrate that the predicted stage shift to more advanced disease at diagnosis of HNC has not been realised in the longer term. This may be because both TNM and AJCC staging are imperfect methods of capturing the true extent of disease burden with recognised limitations. However, other parameters, which may be considered surrogate measures of disease burden, such as symptom duration, patient PS and intent of treatment, were also consistent between the 2 years suggesting that more patients have not presented late with more advanced disease or with non‐radically treatable disease.

However, it is important to note that the majority of patients in both cohorts presented with late‐stage disease, associated with increased morbidity and mortality compared to early stage disease. While stage at diagnosis may not have worsened with the pandemic, we cannot afford to be complacent. Before the COVID‐19 pandemic, several studies had reported poor levels of awareness of red‐flag symptoms and risk factors for HNC.^23^ Public health campaigns to raise awareness of risk factors and red flag symptoms, such as those seen for other cancers,^23^ may be helpful in addressing this long‐standing issue.

This is the first time that patterns of treatment have been evaluated before and after COVID‐19 public health measures. This is important data for healthcare service providers; while the logistics of delivering treatments to patients with HNC has changed with the pandemic, the nature and intent of treatments remain as previously, with no reduction in radical, curative treatments nor increase in palliative approaches. At face value, this is also reassuring for clinicians; however, the true measure of the impact on patients will only be determined when disease control and survival outcomes are known.

The significantly higher number of patients diagnosed in our cancer network between June and October 2021 compared to the equivalent period in 2019 may reflect a backlog of cases which had gone undetected during the most severe restrictions early in 2021 and 2020. At odds with this is that symptom duration reported by patients at the time of diagnosis and the proportion of patients presenting as an emergency were no different between the two cohorts. The apparent increase may simply reflect intrinsic variation in number of diagnoses per month, despite being seasonally matched cohorts.

A concerning finding which is pertinent to the future HNC disease burden was that of a history of alcohol excess in a higher proportion of patients following the pandemic. This trend was also observed by a cross‐sectional study of 21 countries, finding the United Kingdom (but not other European countries) had significantly more alcohol consumption during the pandemic.^24^ The health impact of this in the longer term is yet to be determined.

Limitations of this study are its retrospective nature with data reliant on the accuracy of patient case records. Parameters such as symptom duration, smoking and alcohol history are subject to a potential recall bias and were not consistently recorded. However, objective measures such as staging and treatment delivered, the primary endpoints of our study, are confirmed by MDT discussion and consistently recorded. All patients with a new diagnosis of HNC in our cancer network are entered into the MDT database and so case ascertainment is not subject to selection bias. We acknowledge that our study evaluates the impact of the pandemic in one region and may not be applicable to jurisdictions which were subject to different restrictions. However, our cancer network serves 2.4 million people or 46% of the national population, meaning that our data is likely to be representative of national trends. The immediate impact of the reallocation of healthcare provision and COVID‐19 public health measures internationally on HNC stage at diagnosis is remarkably consistent, with the studies comparing the 2019 and 2020 cohorts described above being carried out in 13 different countries from North America, Europe, Australia and Asia.^5^, ^10^, ^11^, ^12^, ^13^, ^14^, ^15^, ^16^, ^17^, ^18^, ^19^, ^20^, ^21^ The time periods evaluated in our study coincide with the most severe COVID‐19 public health measures being eased in many countries, not just our own.^25^ It is likely therefore, that our study, the first to evaluate the impact of the pandemic on HNC diagnosis and treatment patterns at this later time point, is reflective of the situation internationally.

While these initial findings are reassuring, the true impact of the COVID‐19 pandemic on patients with HNC will remain unclear for many years until long‐term trends are available and survival outcomes are known. More nuanced effects on quality of life and morbidity may be more difficult to evaluate but also warrant further investigation.

## 5 CONCLUSION

For the first time, we demonstrate that the predicted stage shift because of the COVID‐19 pandemic, to more advanced disease at the time of diagnosis of HNC has not been realised in the longer term. In keeping with this, there was no difference in symptom duration, patient PS or treatment patterns between the 2019 and 2021 cohorts.

## AUTHOR CONTRIBUTIONS

Kelten Clements: Data collection; data analysis and interpretation; writing the first draft manuscript. Claire Paterson: Conception and design; critical revision of article. David I. Conway: Conception and design; critical revision of article. Catriona M. Douglas: Conception and design; critical revision of article. Anna Cowell: Conception and design; data collection; critical revision of article. William Flynn: Database construction; data collection; data analysis; critical revision of article. Gillian White: Data collection. All authors read and agreed to the final version of the manuscript.

## CONFLICT OF INTEREST STATEMENT

The authors declare no conflict of interest.

### PEER REVIEW

The peer review history for this article is available at https://www.webofscience.com/api/gateway/wos/peer-review/10.1111/coa.14048.

## ETHICS STATEMENT

This study was approved by the MVLS college ethics committee of the University of Glasgow (Project no: 200210121) for the extraction and analysis of patients' clinical information.

## Supporting information


**TABLE S1.** Univariate analysis of explanatory factors for early/late overall AJCC stage.
**TABLE S2.** Univariate analysis of explanatory factors for treatment intent.

## Data Availability

De‐identified data are available upon reasonable request from the corresponding author.
