# Supplementary material for: The COVID‐19 pandemic has not changed stage at presentation nor treatment patterns of head and neck cancer: A retrospective cohort study
Source: Clin Otolaryngol. 2023 Mar 16;48(4):587–94. doi: 10.1111/coa.14048 (PMC10946702; doi:10.1111/coa.14048)
Supplement: Supplementary file 1 — TABLE S1. Univariate analysis of explanatory factors for early/late overall AJCC stage. TABLE S2. Univariate analysis of explanatory factors for treatment intent. [file COA-48-587-s001.doc]

**Supplimentary Information/Appendix**

***Table S1*** Univariant Analysis of explanatory factors for early/late overall AJCC stage

|  | **Overall AJCC Stage** | | |  |  |
| --- | --- | --- | --- | --- | --- |
| **characteristic** | **Early (I or II)** | **Late (III or IV)** | **overall** | | **P-value** |
| total (%) | 243 (45) | 298 (55) | 541 | |  |
| **demographics** |  |  |  | |  |
| male (% by column) | 170 (70) | 213 (72) | 383 (71) | | 0.700^a^ |
| mean age (SD) | 64 (11.7) | 67 (11.4) | 65 (11.6) | | 0.003^b^ |
| Medium PS (IQR) | 0 (0-1) | 1 (0-2) | 1 (0-2) | | <0.001^c^ |
| Medium SIMD (IQR) | 2 (1-4) | 2 (1-3) | 2 (1-3) | | 0.009^c^ |
| **Risk Factors (%)** |  |  |  | |  |
| Alcohol (%Y) | 76 (31) | 131 (44) | 207 (38) | | 0.003^a^ |
| Smoking (%Y) | 182 (75) | 244 (82) | 426 (79) | | 0.049^a^ |

^a^Mann-Whitney U Test

^b^Spearman Correlation

^c^Kruskal-Wallis H Test

Four cases were excluded from the analysis of stage in the 2021 cohort as the primary tumour could not be assessed

***Table S2*** *Univariant Analysis of explanatory factors for treatment intent*

|  | **Treatment Intent** | |  |  |
| --- | --- | --- | --- | --- |
| **characteristic** | **Palliative** | **Curative** | **overall** | **P-value** |
| total (%) | 184 (34) | 361 (66) | 545 |  |
| **demographics** |  |  |  |  |
| male (% by column) | 126 (69) | 261 (72) | 386 (71) | 0.363^a^ |
| mean age (SD) | 70 (11.2) | 63 (11.0) | 65 (11.6) | <0.001^b^ |
| Medium PS (IQR) | 2 (1-2) | 0 (0-1) | 1 (0-2) | <0.001^c^ |
| Medium SIMD (IQR) | 2 (1-3) | 2 (1-3) | 2 (1-3) | 0.186^c^ |
| **Risk Factors (%)** |  |  |  |  |
| Alcohol (%Y) | 88 (47.8) | 121 (33.5) | 209 (38.3) | <0.001^a^ |
| Smoking (%Y) | 154 (83.7) | 273 (75.6) | 427 (78.3) | 0.031^a^ |

^a^Mann-Whitney U Test

^b^Spearman Correlation

^c^Kruskal-Wallis H Test
